# Benchmarking national action plans on antimicrobial resistance in eight selected LMICs: Focus on the veterinary sector strategies

**DOI:** 10.7189/jogh.10.020414

**Published:** 2020-12

**Authors:** Ebiowei Samuel F Orubu, Indorica Sutradhar, Muhammad H Zaman, Veronika J Wirtz

**Affiliations:** 1 Boston University College of Engineering, Department of Biomedical Engineering, Boston, USA; 2 Boston University Institute for Health System Innovation & Policy, Boston, USA; 3 Boston University School of Public Health, Department of Global Health, Boston, USA

## Abstract

**Background:**

The WHO Global Action Plan on antimicrobial resistance (GAP) provides a global strategy for combating antimicrobial resistance. Context-specific national action plans (NAP) translate GAP to reflect local priorities. However, the process by which countries translate GAP into NAPs, and the resultant concordance, is not well-known. The aim of the paper is to evaluate the NAPs of eight selected low- and lower-middle income countries (LMICs) against GAP and each other to identify best practices with a focus on the veterinary sector.

**Methods:**

Using the WHO GAP, and the WHO Manual for designing NAPs, we performed a policy content evaluation for: Afghanistan, Bangladesh, Ethiopia, Ghana, Nepal, Nigeria, Pakistan and Uganda. NAPs were assessed as concordant with GAP if they contained ≥80% of the recommendations. Operational and monitoring and evaluation (M&E) plans were assessed as: Specific, Measurable, Assignable, and Time-bound (or SMAT). Financing, targets and legislation for antimicrobial use reduction, and medicine quality assurance mechanisms were assessed using a constructed framework. Countries were then ranked using a scoring system to identify best practices.

**Results:**

All NAPs contained ≥80% of GAP’s recommendations. Whereas Nepal’s NAP was strategic, the rest were operational and uniformly SMAT; except Afghanistan’s. The M&E plans were not all SMAT. Detailed costing and funding sources were included for only Ghana and Uganda. Quantitative target for antimicrobial use reduction was found only in Nepal’s NAP and legislation only for Bangladesh. Ghana’s and Uganda’s medicine quality assurance mechanisms were the most robust.

**Conclusions:**

All NAPs were concordant with GAP. However, gaps exist in relation to M&E, diminishing the countries’ capacity to be accountable and implement corrective action if necessary. Most lacked financing plans and targets for antimicrobial use reduction. The antimicrobial quality assurances strategies are limited in most of the NAPs assessed. A mechanism by which countries can benchmark their NAP would allow identification of specific limitations and areas of best practice.

Anti-microbial resistance (AMR), the ability of microbes to withstand treatment with therapeutic doses of antimicrobial agents, is a global public health emergency [1]. It has been estimated that by 2050, AMR may lead to an estimated 300 million human deaths, an 11% drop in livestock production - with consequences for food security, and a loss of about USD 100 trillion, or a 2.5% drop in the global GDP [2,3]. It is possible that by then, AMR would be the leading cause of death – overtaking cancer, cardiovascular diseases and other causes [2]. The greatest impact will be in low- and middle-income countries (LMICs) [3]. Unchecked, AMR has the potential to reverse or prevent the achievement of most of the Sustainable Development Goals (SDGs) [4].

AMR is a natural process driven by many inter-related factors, with the non-evidence based use of antimicrobials in human and animal health a key driver [5-7]. In many countries, the use of antimicrobials in animal populations, mainly for growth promotion or prophylaxis, outstrips human use [2,8]. The World Organization for Animal Health (OIE) estimated the global use of antimicrobials in animals in 2015 at 172 mg/kg [9]. Within livestock, usage differs and ranges from an estimated global average in 2010 of 45 mg/kg for cattle production to 148 mg/kg for poultry and 172 mg/kg for pigs [8]. As the demand for animal protein increases, it is estimated that the non-therapeutic uses of antimicrobial agents in animals, including the use of reserve, or Critically-Important Antimicrobials (CIAs), would increase, especially in countries with significant livestock holdings moving towards intensive food production systems [8,10,11]. Two specific recommendations for combating AMR in the veterinary sector are: (i) restricting the use of CIAs, including in feed; and (ii) reducing the indiscriminate use of antimicrobials in animal production, and limiting all uses to a target maximum 50 mg/kg [11-13]. Thus, global and national, or context-specific, concerted actions are needed to contain AMR.

Globally, efforts to combat AMR in all sectors- human, animal, and environment, are led by the World Health Organization (WHO). In 2015, the WHO, in collaboration with the Food and Agricultural Organization and the OIE, launched the Global Action Plan on antimicrobial resistance (GAP, 2015-2019) containing five strategic objectives for AMR containment [14]. In 2016, the WHO followed up the GAP with a “how-to-do-manual” providing guidance on the development of country-level National Actions Plans (NAPs) [15]. This manual listed three core components of a NAP as: (1) a strategic plan, (2) an operational plan including a financing component, (3) a monitoring and evaluation (M&E) plan. At the United Nations (UN) high commission meeting in 2016, all countries committed to having NAPs, based on the GAP, by 2017. As of March 2019, the WHO reported 67% of the 194 UN member states had a NAP [16].

Context-specific NAPS are needed to effectively combat AMR. However, despite the launch of NAPs in many countries, little is known on how countries are translating GAP into NAPs. Most LMICs also face the problem of poor-quality or substandard and falsified antimicrobials, overall estimated at 10%-20% in the human health sector, with evidence of a similarly high prevalence in the veterinary sector in some settings [17-19]. There is not much information on how countries are addressing medicine quality assurance in the supply chain in the veterinary sector, especially in the context of NAPs on AMR. It has been shown that poor-quality antimicrobials contribute to AMR although the exact mechanisms are unclear [20,21]. There is a need to understand how LMICs with substantial veterinary sectors translate the GAP into NAPs. Additionally, it is necessary to know how the overall process of translating GAP into NAPs may be strengthened, if necessary, to produce context-specific policies to effectively combat AMR. While the WHO tracks self-reported plan implementation, there are scarce records of comparative content analysis of individual plans. Our manuscript seeks to fill this gap.

The aim of this paper was to perform a policy content evaluation of the NAPs of selected LMICs, with a focus on the veterinary sector. The objectives were 2-fold: (1) to benchmark policy provisions against general GAP recommendations in the veterinary sector, and (2) to identify best practices in design of NAPs from benchmarking and a cross-country comparison, with a view to providing lessons for countries designing or iterating NAPs.

## METHODS

### Study design

The study was designed as a policy content evaluation. Two researchers independently evaluated the NAPs on antimicrobial resistance for eight LMICs. Provisions for the veterinary sector in the plans were benchmarked against the WHO GAP for concordance, as detailed in the section on Benchmarking below. In addition, a cross-country comparison of the plans was performed as described in the section titled Cross-country comparison to identify best practices.

### Country selection

Eligibility criteria were low- and lower-middle income countries as defined by the World Bank with a NAP on the WHO database [22,23]. The selection criteria among eligible countries were: size of the livestock economy (selected countries either had high livestock (cattle, poultry, fisheries) holding by population or livestock produce, belonging in each case to the top 2 quartiles of international or regional rankings), and population (**Table 1**). Using these criteria, four countries each from Asia and Africa were selected:

**Table 1:** Demographics and livestock economy of Bangladesh, Pakistan, Ghana, Nigeria, Uganda, Ethiopia, Nepal and Afghanistan included on the basis of an existing NAP. Selected countries either had high livestock (cattle, poultry, fisheries) holding by population or livestock produce, belonging in each case to the top 2 quartiles of international or regional rankings

Country	National Action Plan enacted	Demographic and livestock population					Livestock economy	Source
**Population (million)**	**Population livestock holding (% households)**	**Size of livestock (million)**			**Contribution to GDP, %)**	**Ref.**
				Cattle	Poultry	Others*	Livestock	
Bangladesh	2017	159	80**†**	241	338	315	1.7	[24-26]
Pakistan	2017	180	-	46.1	1200	150.4	11	[27-29]
Ghana	2017	25.4	40	1.66	68.5	11.1	7	[30,31]
Nigeria	2017	190	42	18.4	180	119.4	1.7	[32]
Uganda	2018	40	58	14.2	47.6	24.6	4.3	[33]
Ethiopia	2015	102	70	57	57	53‡	15	[34-36]
Nepal	2016	29.3	70§	7.2	48.5	12	8.4	[37]
Afghanistan||	2017	32.2	68	2.8	13.1	34.4	-	

*Others include sheep, pigs, goats, camels and other animals.

†Rural households.

‡Exact figure varies with data source.

§Population livestock holding figure from 2005.

||Livestock figures for Afghanistan are estimated for 2013-2014 based on census results from 2003.

(I) Lower-middle income: Asia – Bangladesh and Pakistan; Africa – Ghana and Nigeria,

(II) Low-income: Asia – Afghanistan and Nepal; Africa – Uganda and Ethiopia.

Only countries from Asia and Africa were selected because all but five low- and lower-middle income countries are in these regions. The five LMICs outside these regions are in South America and the Caribbean: Bolivia, El Salvador, Haiti, Honduras and Nicaragua, and NAPs were not available for these countries by 2018.

### Benchmarking

**GAP:** Using the GAP framework for national action, the NAP of the eight countries were benchmarked against recommended GAP actions in the veterinary sector. Overall, NAPs were assessed for concordance as previously described [38]. In brief, NAPs were assessed as compliant with GAP if they make policy provisions for at least 80% of the recommended actions for the veterinary sector for strategic objectives 1-4 in the GAP. Benchmarking was performed by a single researcher (SO).

**WHO Manual:** In addition, operational and monitoring and evaluation (M&E) frameworks were assessed if they met four, out of five, SMART criteria: specific, measurable, assignable, and time-bound, as specified in the WHO Manual: the Realistic aspect was excluded because this depends largely on several factors beyond the scope of this paper. Additionally, financing information in the NAP was noted and evaluated for costing and funding source according to the WHO Manual.

### Cross-country comparison

The cross-country comparison was performed by a qualitative assessment of the following parameters:

1.Targets for antimicrobial use reduction in the veterinary sector according to the recommendation of the WHO and specific targets of antimicrobial use reduction as specified in the O’Neill Report, 2015 [12,13].

2. Legislation restricting non-therapeutic uses of antimicrobial for growth promotion, or prophylactically, including CIAs, in the veterinary sector, if any: targets without legislative backing may be difficult to enforce in certain contexts. We include legislation here as an indication of government willingness to enforce targets. This term is also used for policies restricting use in the veterinary sector for growth promotion as specified under Objective 4 of the GAP [14].

3. Medicine quality assurance: The GAP recommends quality assurance of antimicrobial medicines in human and animal health. To operationalize the assessment of the recommendation, we used the medicine supply chain “framework” by Silva et al., 2019. We adapted it to describe nodes at which quality assurance mechanisms would be required [39]. Eight mechanisms covering four nodes and the presence of a National Medicines Regulatory Authority were framed as criteria for assessing the NAPs.

A scoring system was developed using a data extraction tool created on Excel in which scores were tallied for each country using the answers (Yes = 1; No = 0) to questions created for the benchmarking and cross-country comparison parameters (as described above). Countries with the highest tally for each parameter were identified as an example of best practice for the design of National Action Plans on AMR in countries with a similar context.

Evaluation for concordance with the WHO Manual and the cross-country comparisons were independently performed by two researchers (SO & IS). Results were studied for discrepancies and any differences that could not be resolved by the two were referred upwards to VJW and MHZ for resolution.

### Analysis of results

Results are largely descriptive and presented in terms of the scoring system applied.

## RESULTS

### Benchmarking

#### Concordance with GAP

All NAPs were found to be compliant with GAP where all plans had at least 80% of the recommended actions in the veterinary sector for nations in the GAP (**Table 2**). However, most plans lacked diagnosis to guide rational prescription as a recommended action. Some countries showed more gaps in specific areas than others. For example, the NAP of Afghanistan did not include three of the recommended actions within the Infection Prevention and Control (IPC) domain. Similarly, the NAP of Pakistan did not include three actions under the Optimized use of antimicrobials domain. Despite this, within each domain, countries included the majority of GAP recommended actions for each domain.

**Table 2:** Concordance with WHO GAP recommended actions (Objectives 1-4) for all eight selected countries. All countries had scores above 80 showing concordance (“No” indicates policy gaps)

WHO GAP Recommended action for nations	Afghanistan	Bangladesh	Ethiopia	Ghana	Nepal	Nigeria	Pakistan	Uganda
**I. Improve awareness & understanding of AMR:**								
1. Increase national public awareness of AMR through communication	Yes	Yes	Yes	Yes	Yes	Yes	Yes	Yes
2. AMR in professional curricula	Yes	Yes	Yes	Yes	Yes	Yes	Yes	Yes
3. Antimicrobial use & AMR in schools’ curricula	Yes	Yes	Yes	No	Yes	Yes	Yes	Yes
4. AMR in National Risk Register	Yes	Yes	Yes	Yes	Yes	Yes	Yes	Yes
5. One-Health coalitions to address AMR	Yes	No	Yes	Yes	Yes	Yes	No	Yes
**II. Surveillance and research:**								
1. Establish National Reference Centre for data collection and analysis	Yes	Yes	Yes	Yes	Yes	Yes	Yes	No
2. Establish National Reference laboratory (Surveillance)	Yes	Yes	No	Yes	Yes	Yes	Yes	Yes
3. Strengthen surveillance by implementation of guidelines	Yes	Yes	No	No	Yes	Yes	Yes	Yes
4. Share information regionally and globally	Yes	Yes	Yes	Yes	Yes	Yes	Yes	Yes
5. Develop capacities to detect & report emerging resistance	No	Yes	Yes	Yes	Yes	Yes	Yes	Yes
6. Monitor antimicrobial consumption	Yes	Yes	Yes	Yes	Yes	No	Yes	Yes
7. Research to support new treatments	Yes	Yes	Yes	Yes	Yes	Yes	Yes	Yes
**III. Infection Prevention & Control (IPC):**								
1. Urgent action to implement hygiene & IPC	Yes	Yes	Yes	Yes	Yes	Yes	Yes	Yes
2. Hygiene and IPC in curriculum	Yes	Yes	Yes	No	Yes	Yes	Yes	Yes
3. Strengthen IPC policies & SOPs in HCF; M&E	No	Yes	Yes	Yes	Yes	Yes	Yes	Yes
4. Antimicrobial sensitivity data	No	Yes	Yes	Yes	Yes	Yes	No	Yes
5. Animal health practices compliance with OIE and FAO/WHO codex	Yes	Yes	No	No	Yes	Yes	Yes	Yes
6. Vaccination	No	Yes	Yes	Yes	Yes	Yes	Yes	Yes
**IV. Optimized use of antimicrobials:**								
1. Distribution, prescribing and dispensing on license	Yes	Yes	Yes	Yes	Yes	Yes	Yes	Yes
2. License only quality-assured antimicrobials	Yes	Yes	Yes	Yes	No	Yes	No	Yes
3. EML & STGs; regulation of promotion	Yes	Yes	Yes	Yes	Yes	Yes	Yes	Yes
4. Diagnosis to guide rational prescription	No	Yes	Yes	Yes	Yes	No	No	Yes
5. Antimicrobial Stewardship at national and local levels	Yes	Yes	Yes	Yes	Yes	Yes	Yes	Yes
6. Encourage appropriate antimicrobial use	Yes	Yes	Yes	Yes	Yes	Yes	No	Yes
7. Governance of supply chain for antimicrobial agents	Yes	Yes	Yes	Yes	Yes	Yes	Yes	Yes
8.Eliminate non-therapeutic uses of antimicrobials in animals	Yes	Yes	Yes	No	Yes	Yes	Yes	Yes
**Score for concordance with GAP**	**81**	**96**	**88**	**81**	**96**	**92**	**81**	**96**

#### Concordance with the WHO Manual

**Operational plan:** Operational plans were assessed as specific, measurable, assignable and time-bound, but for Afghanistan’s and Nepal’s (**Table 3**). Nepal did not include an operational plan, only a strategic one. Of the other seven countries, only Afghanistan’s was assessed as not time-bound.

**Table 3:** Cross-country comparison of NAPs of eight selected low- and lower-middle income countries against 16 criteria in five domains derived from international recommendations or guidelines

Criterion	Afghanistan	Bangladesh	Ethiopia	Ghana	Nepal	Nigeria	Pakistan	Uganda
**1. Target** for antimicrobial use reduction								
i. Is there a **quantitative** target?	No	No	No	No	Yes	No	No	No
ii. Any planned intervention(s) to reduce **non-therapeutic antibiotic** uses?	Yes	Yes	Yes	No	Yes	Yes	Yes	Yes
iii. Any actions planned to reduce the use of reserve or **Critically Important Antimicrobials** (CIA)?	Yes	Yes	Yes	No	Yes	No	No	No
iv. Any planned intervention to ban the use of antibiotic-containing **feeds***?*	No	Yes	Yes	No	Yes	Yes	No	Yes
**2. Legislation** restricting uses of antimicrobials for growth promotion or disease prevention in livestock?	No	Yes	No	No	No	No	No	No
**3. Antimicrobial quality** assurance strategies								
i. Is there a program/intervention assuring the quality of antimicrobials?	Yes	Yes	Yes	Yes	Yes	Yes	Yes	Yes
ii. Is there a separate veterinary medicines regulatory agency for the quality control of antimicrobials used in animals?	No	No	Yes	No	No	No	No	No
**4. Operational plan**								
• Specific	Yes	Yes	Yes	Yes	No	Yes	Yes	Yes
• Measurable	Yes	Yes	Yes	Yes	No	Yes	Yes	Yes
• Assignable	Yes	Yes	Yes	Yes	No	Yes	Yes	Yes
• Time-bound	No	Yes	Yes	Yes	No	Yes	Yes	Yes
**5. Monitoring and Evaluation** (M&E) **plan**:								
• Is there a separate M&E plan?	Yes	No	No	Yes	No	Yes	Yes	Yes
• Specific	Yes	No	Yes	Yes	No	Yes	Yes	Yes
• Measurable	Yes	No	No	Yes	No	Yes	Yes	Yes
• Assignable	No	No	Yes	Yes	No	No	Yes	No
• Time-bound	Yes	No	Yes	Yes	No	Yes	Yes	Yes
**6. Score across the 16 criteria**	**10**	**9**	**12**	**10**	**5**	**11**	**11**	**11**

**Monitoring and Evaluation (M&E) plan:** Most (5/8, 63%) of the NAPs included a separate M&E plan (**Table 3**). Bangladesh, Ethiopia and Nepal did not include a separate M&E section. While Bangladesh and Ethiopia’s NAPs had integrated M&E actions, Nepal’s did not include any M&E. However, while Bangladesh’s integrated M&E activities were assessed as neither specific, measurable nor time-bound, Ethiopia’s was assessed as being only not measurable. M&E plans were not assignable for five countries: Afghanistan, Bangladesh, Nepal, Nigeria, and Uganda.

**Financing:** Of the eight LMICs assessed, costing for each activity was included only for Afghanistan, Ghana and Uganda (**Table 4**). Funding sources were indicated for only three countries ie, Ghana, Nigeria and Uganda. Afghanistan and Nigeria included partial information on funding. Ethiopia's NAP makes it clear that its proposed activities would first need to be prioritized before funding would be decided. Financing, where indicated, included external funding sources such as WHO.

**Table 4:** Financing information contained in the National Action Plans of the evaluated countries

Questions/Queries	Afghanistan	Bangladesh	Ethiopia	Ghana	Nepal	Nigeria	Pakistan	Uganda
Is each proposed activity **costed**?	Yes	No	No	Yes	No	No	No	Yes
Is the **source(s)** of funding indicated?	No	No	No	Yes	No	Yes	No	Yes
Sources*	WHO (for only two activities related to creating awareness under objective 1). For all other activities, there is no indication of source.			Government; corporate institutions; development partners; non-governmental organizations.		Government, donor agencies, development partners		Government/partners (meaning international development partners such as WHO)

*These are indicated as possible sources of funds. In some cases, there sources actively fund some activities, for example funding by WHO for the Antimicrobial Awareness Week programs in many countries. Inclusion here does not imply active funding by the common funding sources included here.

### Cross-country comparison

**Targets for antimicrobial use reduction**: Interventions to reduce veterinary antimicrobial use were included as plans in all NAPs, except Ghana’s. However, quantitative targets are included only in Nepal's. Half (50%) of the NAPs made no specific mention of reserve antimicrobials or CIAs as defined by WHO (11). In many cases, CIAs are included under the general antimicrobial category. Only Afghanistan, Bangladesh, Ethiopia, and Nepal expressly mention CIAs. Similarly, only 50% of the NAPs include planned interventions to ban the use of antibiotic-containing feeds.

**Legislation**: Legislation restricting the non-therapeutic uses of antimicrobials in livestock was found only in Bangladesh. However, there were indications of intention to make these legislations either de novo or by modification of existing legislation eg, in Pakistan. Only the NAP of Ethiopia mentions a separate veterinary agency that is assumed to monitor or ensure the quality of veterinary medicines. The NAP of Pakistan mentions a separate veterinary agency for monitoring quality, but it is not known if this body also has authority over veterinary antimicrobials.

**Antimicrobial quality assurance strategies**: All NAPs contained, to varying degrees, mechanisms for the quality assurance of antimicrobials (**Table 5**). Only two countries met all specified criteria: Ghana and Uganda. Bangladesh and Ethiopia met all but one. Nepal’s NAP was a strategic document without detailed operational plans.

**Table 5:** Cross-country assessments of NAPs provision for medicine quality assurance using criteria targeting pharmaceutical supply chain nodes derived from a generic model*†

	Criterion	Afghanistan	Bangladesh	Ethiopia	Ghana	Nepal	Nigeria	Pakistan	Uganda
1	Is there a National Medicines Regulatory Agency (NMRA)?	Yes	Yes	Yes	Yes	Yes	Yes	Yes	Yes
2	Does the NMRA oversee quality of antimicrobials?	Yes	Yes	Yes	Yes	Yes	Yes	Yes	Yes
3	Is there any mention of Good Manufacturing Practices for manufacturers?	No	Yes	Yes	Yes	No	Yes	No	Yes
4	Are importers/wholesalers/suppliers required to comply with quality checks?	Yes	Yes	Yes	Yes	No	No	No	Yes
5	Any mention of Good Pharmacy Practice, or antimicrobials as Prescription Only Medicines?	Yes	Yes	Yes	Yes	Yes	Yes	Yes	Yes
6	Procurement/sourcing – Quality-assured antimicrobials or good procurement practices including quality?	Yes	No	Yes	Yes	No	Yes	No	Yes
7	Any mention of proper storage conditions for antimicrobials?	No	Yes	No	Yes	No	No	No	Yes
8	Any mention of post-marketing surveillance for quality?	Yes	Yes	Yes	Yes	No	Yes	Yes	Yes
	**Score for quality assurance mechanism**	**6**	**7**	**7**	**8**	**3**	**6**	**4**	**8**

*Criteria are derived from professional practice experience, and are not exhaustive.

†Scores are tallied for each country at the bottom of the table. Yes indicates presence, and No indicates absence, of the criterion from the NAP. Of all countries assessed, only Ghana and Uganda met all criteria, indicating robust NAP provisions for medicine quality assurance. Nepal’s plan was a strategic document. Of all countries with operational plans, Pakistan’s NAP had the lowest quality score of 4.

## DISCUSSION

The WHO Global Action Plan on antimicrobial resistance represents the first global tool for the containment of AMR. Over time, this has been followed by several tools including the Manual for Developing NAPs [40]. A NAP, as it maps a committed plan of action agreed by stakeholders, is an essential tool in combating AMR in different national settings. There is a need for this plan to be comprehensive (ie, include all considerations for tackling AMR), and for plan implementation to be continuously evaluated, and publicly reported. This evaluation benchmarked and compared the NAP of eight selected LMICs with a significant livestock economy against the WHO’s GAP and Manual, and other parameters derived from the literature on AMR, or constructed, to identify gaps and best practices in the design of NAPs. Such an evaluation can provide useful insights for policy-makers in countries with similar settings that are designing or iterating NAPs. To the best of our knowledge, this is the first of such focused independent policy content evaluation.

This evaluation demonstrates that indeed all the studied NAPs used the GAP as a template. Without exception, all had at least 80% of all recommended actions for veterinary antimicrobial resistance containment. This was regardless of whether the NAP was a broad strategy document, as for Nepal, or included a detailed implementation or operational scheme as for all other included countries.

However, NAPs differed in terms of operational, including financial, and M&E plans as outlined in the WHO Manual; as well as in targets and legislation for antimicrobial use reduction, and medicine quality assurance mechanisms. We found that the operational plans for all countries except Afghanistan and Nepal were uniformly detailed. However, many countries have gaps in their M&E plans. Countries whose NAPs do not include an M&E framework, or whose M&E framework are not sufficiently robust could use the plans of countries such as Ghana and Pakistan as models of best practice, or could use the WHO Monitoring and Evaluation framework to design one [41]. It may also be useful if such evaluations can be made publicly available as suggested and practiced by the United States [42,43]. The WHO maintains a database of self-reported progress in the implementation of NAPs for countries [23].

Of all the countries only Nepal’s NAP contained a target for antimicrobial use reduction. In high-income countries with advanced surveillance systems, antimicrobial usage is monitored via sales or consumption data with policies designed to reduce use yielding positive results. Recently, the consumption of antimicrobials in food-production animals dropped by 32% across the European Union [44]. For most of these countries, there were/are established targets for use reduction. In Asia, the NAP of Thailand includes a target of 30% reduction in veterinary antimicrobial usage by 2021 [45]. The absence of use reduction targets in the NAP of most of the studied LMICs needs to be addressed. Targets should be included for both all antimicrobials and specifically for reserve antimicrobials or CIAs [42]. To define country-specific targets it is important to have baseline data on usage. Prospectively, countries will need to develop surveillance systems to routinely collect data on consumption.

Similarly, the lack of legislation restricting the non-therapeutic use of antimicrobials in animals needs to be addressed. It is possible that some countries such as Pakistan are in the process of doing so. It would be necessary for countries to have legislation restricting the use of antimicrobials in animals and to include these in the NAP – with incentives, alternatives and disincentives to guide farmers, veterinarians and other stakeholders. This should also be the case with antimicrobial-containing feeds. Interventions to eliminate antimicrobials from feed would also be necessary. Countries can learn from Bangladesh’s NAP which contains three legislations restricting non-therapeutic uses in animals [46].

Antimicrobial quality assurance strategies in the NAPs of the selected countries need to be strengthened. Poor-quality veterinary antimicrobials are prevalent in Ethiopia and Nigeria, for example. All countries differed on the quality score from a low score of 3 (Nepal) and 4 (Pakistan) to a high of 8 (Ghana and Uganda). The other countries had high scores of between 6 and 7. There is a need to make provisions for assuring the quality of antimicrobials throughout the supply chain. Ghana’s and Uganda’s NAP may be used as best practices in this regard. A stringent regulatory authority is one prerequisite for ensuring the quality of medicines throughout the supply chain for human and veterinary medicines [19]. Of all the studied countries, only Ethiopia had a separate agency for the control of veterinary medicines quality [47]. While the existence of a separate regulatory agency may not by itself lead to the elimination of poor-quality veterinary medicines from circulation, it does represent capacity advance in veterinary medicines regulation. A separate veterinary medicines regulatory authority also recognizes the one health approach to AMR containment.

Financing for NAP activities is poorly defined for most of the studied LMICs, with all but Afghanistan, Ghana and Uganda not including costing for proposed activities. Overall, this finding agrees with a study by Shabangu et al, 2019, who found that budgets were included in only 19% of 43 NAPs [48]. A previous study of Bangladesh showed that some veterinary-related activities are funded under other programs (the SDGs) - implying a measure of financing for these activities [38]. It should be noted though that Afghanistan like Ethiopia indicates that its proposed activities need to be prioritized before financing is decided. Thus, whilst all countries seem to recognize the need for funding NAP activities, funding decisions still need to be finalized in some countries. The lack of a clear funding structure in the NAP may be a barrier to successful implementation.

Additionally, while financing for national action plans is recognized as important, information as to total costs of implementation is scarce for many LMICs. Ghana estimated that implementation of its 5-year, 2017-2021, national action plan would cost about $21 million [49]. In the United States, funding to implement activities as outlined in the U.S National Action Plan for combating antibiotic resistance activities have been consistently specified, and for the fiscal year 2020 was $170 million [50].

Instituting a cross-country mechanism for making a similar, but more rigorous evaluation of NAP design, may contribute to knowledge, and help countries designing or iterating national action plans [42,51].

### Limitations

This evaluation only considered the presence or absence of provisions for the veterinary sector in the national action plans at a high level. It did not examine the adequacy of the provision, nor does it attempt to imply that this analysis provides an indication of willingness or capacity to implement these provisions. Second, it does not take into recognition the diversity of the political contexts, for example stability of governments, in these countries. Third, we decided not to use a weighted system. There is no gold standard on how to weigh different strategies regarding their priority in the national action plan. These differences need to be accounted for in a more comprehensive study assessing the country-specific needs and capacities which was beyond the scope of this evaluation.

## CONCLUSION

The NAPs of Afghanistan, Bangladesh, Ethiopia, Ghana, Nepal, Nigeria, Pakistan and Uganda – LMICs with significant livestock economies – contained 80% of the WHO GAP recommended actions. However, compared against each other, there are gaps in key areas such as finance, targets and legislation for reducing antimicrobial use in the veterinary sector and medicine quality assurance. Despite this, best practices were identified that can help strengthen the design of NAPs. In addition to efforts by the WHO, a cross-country mechanism by which countries compare NAPs may be a useful tool in the global drive to combat AMR.
